# Switching Between Methanol Accumulation and Cell Growth by Expression Control of Methanol Dehydrogenase in *Methylosinus trichosporium* OB3b Mutant

**DOI:** 10.3389/fmicb.2021.639266

**Published:** 2021-03-22

**Authors:** Hidehiro Ito, Kosei Yoshimori, Masahito Ishikawa, Katsutoshi Hori, Toshiaki Kamachi

**Affiliations:** ^1^Department of Life Science and Technology, Tokyo Institute of Technology, Tokyo, Japan; ^2^Department of Biomolecular Engineering, Nagoya University, Nagoya, Japan

**Keywords:** methanotroph, methane, methanol, methane monooxygenase, methanol dehydrogenase

## Abstract

Methanotrophs have been used to convert methane to methanol at ambient temperature and pressure. In order to accumulate methanol using methanotrophs, methanol dehydrogenase (MDH) must be downregulated as it consumes methanol. Here, we describe a methanol production system wherein MDH expression is controlled by using methanotroph mutants. We used the MxaF knockout mutant of *Methylosinus trichosporium* OB3b. It could only grow with MDH (XoxF) which has a cerium ion in its active site and is only expressed by bacteria in media containing cerium ions. In the presence of 0 μM copper ion and 25 μM cerium ion, the mutant grew normally. Under conditions conducive to methanol production (10 μM copper ion and 0 μM cerium ion), cell growth was inhibited and methanol accumulated (2.6 μmol·mg^−1^ dry cell weight·h^−1^). The conversion efficiency of the accumulated methanol to the total amount of methane added to the reaction system was ~0.3%. The aforementioned conditions were repeatedly alternated by modulating the metal ion composition of the bacterial growth medium.

## Introduction

Methane is a principal component of natural and shale gases. As its global reserves are vast, it is regarded as a next-generation carbon feedstock. Biogas produced by microbial biomass digestion under anaerobic conditions is composed mainly of methane. Microbial methane fermentation can use carbon dioxide as a carbon source. Therefore, methane is expected to be a valuable industrial fuel source as it is renewable and the technologies associated with its collection, production, and consumption are sustainable. Nevertheless, its global warming potential is 25× higher than that of carbon dioxide [U.S. Environmental Protection Agency (EPA), 2019]. Hence, it is a prime mitigation target in greenhouse gas reduction (Shindell et al., 2012). A major source of atmospheric methane is the anaerobic decomposition of biomass from anthropogenic industrial waste production and agricultural activity (Conrad, 2009). However, the methane produced by archaea is low concentration and the collectable gas contains impurities such as carbon dioxide. Thus, it can only be used as fuel without any purification. In contrast, methanol is readily available as a carbon feedstock for further chemical conversion and is easy to store and transport because it is a liquid. Therefore, the efficient conversion of methane to methanol may help increase the availability of heretofore underutilized methane resources.

As methanotrophs are powerful oxidizers, their exploitation in the industrial oxidation of methane to methanol has been investigated. Aerobic methanotrophs oxidize methane to methanol *via* methane monooxygenase (MMO) and NADH at ambient temperature and pressure. Thence, the methanol is oxidized to formaldehyde by methanol dehydrogenase (MDH). The formaldehyde may either be assimilated into biomass or oxidized to formate by formaldehyde dehydrogenase (FADH). The formate is then converted to carbon dioxide *via* formate dehydrogenase (FDH), the NADH is regenerated for MMO, and the cycle is completed. The simplest and most cost-effective approach to the foregoing process is the use of whole-cell methanotroph cultures (Bjorck et al., 2018). Whole cells undergo autopoiesis by biosynthesizing enzymes and replicating themselves. Moreover, the methanol biosynthesized by whole cells can be easily separated. In methanol biosynthesis *via* MMO and methane oxidation, reducing equivalents are required. Whole-cell methanotrophs can supply the reducing equivalent in the form of NADH. A major problem with the application of whole-cell methanotrophs for methanol production is that the microorganisms continue to oxidize the methanol to formaldehyde and thence to formate. Therefore, methanol oxidation must be interrupted so that the methanol can accumulate. The irreversible MDH inhibitor cyclopropanol binds pyrroloquinoline quinone (PQQ) and deactivates MDH (Frank et al., 1989; Shimoda and Okura, 1990; Takeguchi et al., 1997; Furuto et al., 1999). High phosphate or sodium chloride concentrations in the medium also promote methanol accumulation (Mehta et al., 1987; Cox et al., 1992; Dales and Anthony, 1995; Lee et al., 2004; Duan et al., 2011; Han et al., 2013). However, complete MDH inhibition arrests cell growth and the loss of reducing power it causes impedes the natural biochemical pathways of the cell. For efficient methanol production by MDH inhibition, external electron donors such as formate must be added to regenerate NADH (Mehta et al., 1991; Furuto et al., 1999; Lee et al., 2004; Kim et al., 2010; Duan et al., 2011; Pen et al., 2014, 2016). Even when these are supplied, however, the methanotrophs cannot be reused as their MMO activity is destabilized. Hence, the strategic design of methanol production and cell growth with enzyme recovery and reducing equivalents is necessary for continuous methanol production with whole-cell methanotrophs. In this process, it is important to control MDH activity in the bacteria. Lowering MDH activity causes methanol to accumulate, restores MDH activity, and provides intracellular reducing power. However, it is difficult to modulate intracellular MDH activity *via* conventional inhibitor-based methods.

To solve this problem, we examined gene regulation systems in methanotrophs in response to certain metal ions. The canonical “copper switch” controls the expression of two types of MMO (Yoon et al., 2010). Particulate MMO (pMMO) is localized to the intracytoplasmic membranes and its expression and activity increase with copper ion availability. In contrast, soluble MMO (sMMO) occurs in the cytoplasm and is only expressed in the absence of copper ions. MxaF and XoxF are forms of the main component of PQQ-dependent MDH and are found in certain methanotrophs such as *Methylosinus trichosporium* OB3b. MxaF has a calcium ion in its active site (Williams et al., 2005) whereas the active site of XoxF has a rare earth element such as a cerium, lanthanum, or praseodymium ion (Hibi et al., 2011; Nakagawa et al., 2012; Keltjens et al., 2014; Pol et al., 2014). Rare earth elements are gene-regulating factors and control MxaF and XoxF expression. Farhan Ul Haque et al. (2015) first reported that *mxaF* and *xoxF* expression in *M. trichosporium* OB3b substantially decreased and increased, respectively, with increasing cerium ion concentration. This mechanism is known as the “lanthanide switch” (Vu et al., 2016; Groom et al., 2019). Therefore, this lanthanide switch could effectively control MDH activity in whole-cell methanotrophs.

**Figure 1:**
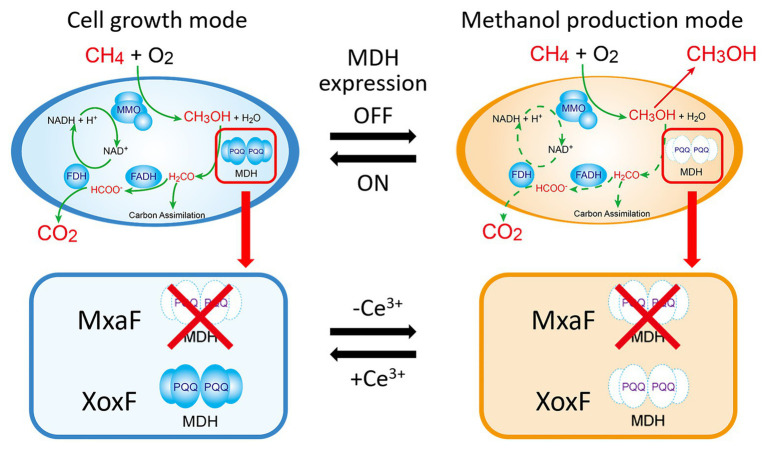
Cell growth and methanol production induced in methanotrophs by switching methanol dehydrogenase gene expression. Solid green line in the cell growth mode means the metabolic pathway of methane in the presence of cerium ions. Dotted green line in the methanol production mode means that the pathway is blocked in the absence of cerium ions. MMO: methane monooxygenase; MDH: methanol dehydrogenase; FADH: formaldehyde dehydrogenase; FDH: formate dehydrogenase; PQQ: pyrroloquinoline quinone.

Here, we used the MxaF knockout mutant of *M. trichosporium* OB3b to test the aforementioned strategic methanol production and cell growth design (Figure 1). This mutant was constructed by referring to the methods of previous studies (Semrau et al., 2013; Farhan Ul Haque et al., 2016). This mutant can only use XoxF MDH for methanol oxidation. Hence, XoxF MDH downregulation is expected to result in methanol generation in the absence of cerium. This mutant can again resume growth back by re-expression of XoxF MDH in the presence of cerium. In addition, NADH recycling and/or MMO refreshing through the methanol metabolic pathway restoration may be achieved. Using the lanthanide switch over a long methanol production period without the addition of an external electron source. In this study, we reported switching of methanol production and cell growth in this mutant by modulating the copper and cerium ion content in the growth medium.

## Materials and Methods

### Microbial Growth Conditions

Nitrate mineral salt (NMS) medium (Whittenbury et al., 1970) was used to grow wild type *M. trichosporium* OB3b and the *mxaF* knockout mutant (*ΔmxaF*, Table 1). Methane was the sole carbon and energy source in the growth substrate. Cultures were supplemented with copper ion (as CuSO_4_) and/or cerium ion (as CeCl_3_) from stock solutions prepared in ultrapure water. Copper or cerium ion stock solutions were sterilized by filtration through polyvinylidene fluoride (PDVF) membranes with 0.22-μm pore size. *Methylosinus trichosporium* OB3b wild type and *ΔmxaF* cultures were grown at 30°C with constant shaking at 130 rpm and under a 1:4 (v/v) methane/air mixture in 30 ml NMS in 200-ml Erlenmeyer flasks fitted with baffles. All reagents were obtained from commercial suppliers and were of the highest available purity.

**Table 1:** Bacterial strains and plasmids used in this study.

Strain or plasmid	Description	Reference or source
***Methylosinus trichosporium* OB3b**		
Wild type	Wild type strain	ATCC35070
SC-SacB	Single-crossover mutant constructed by integration of pK18mobsacB_UpDw in flanking region of *mxaF* of *M. trichosporium* OB3b	In this work
OB3b *ΔmxaF*	Unmarked mutant of *mxaF* (METTRDRAFT_RS0210410)	In this work
***Escherichia coli***		
Turbo	*F' proA+B+lacIq ΔlacZM15/fhuA2 Δ(lac-proAB) glnV galK16 galE15 R(zgb-210::Tn10) Tets endA1 thi-1 Δ(hsdS-mcrB)5*	NEB product info.
S17-1	*recA1 thi pro hsd*R-RP4-2Tc::Mu Km:Tn7	Simon, 1984
**Plasmids**		
pJQ200SK	Suicide vector, *P15A*, *traJ*, *oriT*, *sacB*, Gm^r^	Quandt and Hynes, 1993
pJQ200SK_UpMxaDw	DNA fragment containing from upstream to downstream regions of *mxaF* ligated into *Bam*HI site of pJQ200SK	In this work
pJQ200SK_UpDw	DNA fragment containing upstream and downstream regions of *mxaF* ligated into *Bam*HI site of pJQ200sk	In this work
pK18mobsacB	Suicide vector, *oriT*, Km^r^, *lacZ*, *sacB*	Schäfer et al., 1994
pK18mobsacB_UpDw	DNA fragment containing upstream and downstream regions of *mxaF* ligated into *Bam*HI site of pK18mobsacB	In this work

Km^r^, kanamycin resistance; Gm^r^, gentamicin resistance.

A starter culture grown in NMS with 25 μM cerium ion was used as an inoculum to examine the growth of the OB3b *ΔmxaF* mutant in the presence of copper and cerium ions. At the late exponential phase, the cultures were harvested by centrifugation at 3,000 × g and 4°C for 10 min. The cell pellets were washed twice in NMS free of copper and cerium. After each washing, the pellets were resuspended in an equal volume of NMS free of copper and cerium and centrifuged at 3,000 × g and 4°C for 10 min. After the second washing, the cell pellets were resuspended in NMS and inoculated into new cultures in 50-ml serum bottles each containing 10 ml NMS. The initial target OD_540_ was 0.1. Four different culture conditions were considered and cell growth and methanol production were measured under various metal ions concentrations. The bottles were sealed with rubber stoppers and aluminum caps and the headspaces were filled with a 1:4 (v/v) methane/air mixture. The serum bottles were incubated at 30°C and 130 rpm in a shaking incubator (BR-43FL·MR; TAITEC, Saitama, Japan). At the stationary phase, the cultures were harvested by centrifugation at 3,000 × g and 4°C for 10 min and washed twice under the aforementioned conditions. In one experiment, cell pellets were transferred to 50-ml serum bottles each containing 10 ml identical fresh medium and the initial target OD_540_ was 0.1 (transfer A). In another experiment, cell pellets were transferred to 50-ml serum bottles each containing 10 ml identical fresh medium (transfer B). To toggle between cell growth and methanol production, the initial medium OD_540_ was adjusted to 0.1 after inoculation.

*Escherichia coli* were grown in Luria-Bertani (LB) medium. Gentamicin (10 μg ml^−1^) or kanamycin (20 μg ml^−1^) was used to conserve *E. coli* containing mutant construct plasmids and *M. trichosporium* OB3b mutant cultures after the first homologous recombination.

### Construction of the *Methylosinus trichosporium* OB3b *ΔmxaF* Mutant

The double homologous recombination method was used to construct the OB3b *ΔmxaF* mutant by referring to the methods of previous studies (Supplementary Figure S1; Semrau et al., 2013; Farhan Ul Haque et al., 2016). Briefly, pJQ200SK and pk18mobsacB plasmids were amplified in *E. coli* Turbo by the Inoue method (Sambrook and Russell, 2006). Two DNA fragments ~1 kb distant from the 5' to 3' ends (arms A and B, respectively) of *mxaF* in *M. trichosporium* OB3b were amplified by PCR. Specific primers (Table 2) were designed to amplify arms A and B, introduce the *Bam*HI restriction site sequences, and facilitate directional cloning into the pJQ200SK and pk18mobsacB suicide vectors. The PCR products of arms A and B were gel-purified with a QIAquick gel extraction kit (Qiagen, Valencia, CA, United States) following the manufacturer’s instructions. The pJQ200SK plasmid was digested with *Bam*HI and gel-purified with a QIAquick gel extraction kit. The PCR product and linearized pJQ200SK were ligated with an In-Fusion cloning kit (TaKaRa Bio Inc., Kusatsu, Shiga, Japan) following the manufacturer’s instructions. Then *mxaF* in the pJQ200SK_UpMxaDw plasmid was removed with PrimeStar Max DNA polymerase (TaKaRa Bio Inc., Kusatsu, Shiga, Japan) following the deletion protocol. The resulting plasmid was named pJQ200SK_UpDw and digested with *Bam*HI. The connected regions upstream and downstream from *mxaF* (UpDw arm) were gel-purified with a QIAquick gel extraction kit. The pk18mobsacB plasmid was digested with *Bam*HI and gel-purified with a QIAquick gel extraction kit. The UpDw arm and linearized pk18mobsacB were ligated with a ligation-convenience kit (NIPPON GENE, Toyama, Japan). The resulting plasmid was the final construct. It was named pK18mobsacB_UpDw, transferred to chemically competent *E. coli* S17.1 λpir (Simon, 1984) by the heat shock method, and transferred to *M. trichosporium* OB3b *via* conjugation between the donor (*E. coli* S17.1 λpir containing the pK18mobsacB_UpDw construct) and the recipient (*M. trichosporium* OB3b) strains according to previously described methods (Martin and Murrell, 1995; Farhan Ul Haque et al., 2016). The mixture (1:5 donor:recipient ratio at OD_540_) was passed through a 0.2-μm nitrocellulose filter (ADVANTEC Co. Ltd., Tokyo, Japan). The obtained filter was then incubated at 30°C on NMS agar containing 0.02% (w/v) proteose peptone under a 50:50 (v/v) methane/air atmosphere for 24 h. The cells were removed by vortexing the filter in 1 ml NMS medium. The suspension was spread onto NMS agar plates containing 10 μg ml^−1^ kanamycin to select for *M. trichosporium* OB3b harboring the kanamycin resistance cassette and other pK18mobsacB_UpDw regions and 10 μg ml^−1^ nalidixic acid to prevent *E. coli* growth. The NMS agar plates were incubated at 30°C under a 50:50 (v/v) methane/air atmosphere for 2–3 weeks. The *M. trichosporium* OB3b colonies (first homologous recombinants; *M. trichosporium* OB3b SC-SacB) were suspended in NMS and inoculated onto NMS agar plates containing 0.5% (w/v) sucrose to select for *M. trichosporium* OB3b without the *sacB* regions and 25 μM cerium to express XoxF instead of MxaF. The NMS agar plates were incubated at 30°C under a 50:50 (v/v) methane/air atmosphere for 10 days. The *M. trichosporium* OB3b colonies (second homologous recombinants; *M. trichosporium* OB3b *ΔmxaF* mutant) obtained were suspended in NMS containing 25 μM cerium. PCR and sequencing analysis confirmed success of the double homologous recombinants and crossover mutant genotypes.

**Table 2:** Primers used in this study.

Name	Sequence 5'>3'
Nup-F (armA)	CAGCCCGGGGGATCCAATGTGGGTTTCGGTC
Ndown-R (armB)	AGAACTAGTGGATCCTCAATGAGCGAGCG
DLmxaF-F	TCGTCGATAACTGACCCTTCGCCGC
DLmxaF-R	GGTCAGTTATCGACGAGTCCTCCTGC
mxaF_upst-F1 (P1)	CACCATTTCGCCTATCGTCTCAATC
mxaF_dwst-R1 (P2)	CCGGTGACCGTGTTGTGAAAG
mxaF-F2 (P3)	TACCGAGCTCGAATTCAGGAGGACTCGTCGATGAGGAAG
mxaF_R2 (P4)	ACGGCCAGTGAATTCTCAGTTCGCCTTGTACTCGCC
mxaF_mid-F	AAGGGCGTCGAATATGTCGCGATCCTGTAC
mxaF_mid-R	TTCGTCAGAGCCTCGAGCTTGTCCTC
mxaF_upst-F2	GGCGAGAATATTGGCGTAGGCCGGATAG
mxaF_dwst-R2	TGGGGTCATGCTTGGGATCGTATTTCGAG
pK18mobsacB_seq-F	AAGCCCACTGCAAGCTACCTG
pK18mobsacB_seq-R	ACCTACACCGAACTGAGATACCTAC

*Underlined sequences represent different restriction sites*.

### Methanol Concentration

Methanol concentrations were measured by a gas chromatograph (GC-2014; Shimadzu Corp., Kyoto, Japan) fitted with a flame ionization detector (FID) and a packed glass column (2 m × 3 mm i.d.; GL Science, Tokyo, Japan) containing sorbitol 25%-gasport B (60/80 mesh; GL Science, Tokyo, Japan). Nitrogen was the carrier gas and the flow rate was 40 ml/min. The operating temperatures were injection, 150°C; column, 100°C; and detector, 150°C. Five microliters sample solution was injected for each measurement.

### Immunodetection

Anti-XoxF and anti-MxaF antisera were generated against synthesized peptides corresponding to residues 339–351 of MxaF and residues 374–387 of XoxF1, respectively. Whole-cell lysates of *M. trichosporium* OB3b and OB3b *ΔmxaF* mutant were separated on 10% (w/v) acrylamide gel and transferred to a PVDF membrane following general protocols. The Precision Plus Protein™ Dual Color Standards (Bio-Rad Laboratories, Hercules, CA, United States) were used for the molecular weight marker. The blotted membrane was blocked with 5% (w/v) skim milk at room temperature for 1 h and treated at room temperature for 1 h with anti-MxaF or anti-XoxF1 antisera at 1:2,000 dilution in Tris-buffered saline (TBS) containing 0.05% (v/v) Tween 20 (TBS-T; Calbiochem, San Diego, CA, United States). Membrane-bound MxaF and XoxF1 were detected with horseradish peroxidase-conjugated anti-rabbit IgG antibody (GE Healthcare, Little Chalfont, United Kingdom) at 1:10,000 dilution in TBS-T and visualized using EzWestLumi Plus (ATTO Corp., Tokyo, Japan) according to the manufacturer’s instructions.

## Results

### Construction of *Methylosinus trichosporium* OB3b *ΔmxaF* Mutant

**Figure 2:**
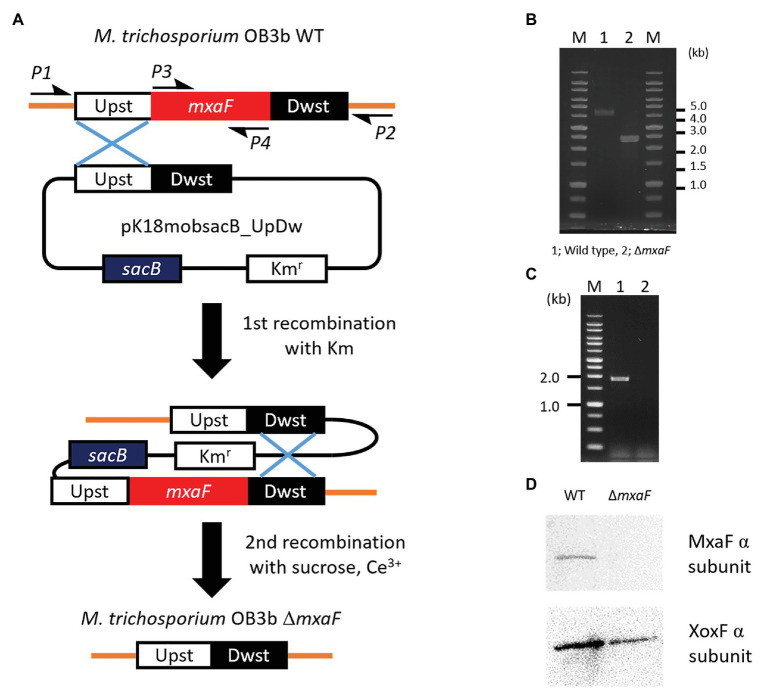
Plasmid-based counterselection. **(A)**. Suicide plasmids used *Km^r^* (kanamicine resistance gene) and *sacB* as counterselectable markers. Half arrows indicate primers (P1, mxaF_upst-F1; P2, mxaF_dwst-R1; P3, mxaF-F; P4, mxaF-R). Primer nucleotide sequences are shown in Table 2. WT, wild type; *ΔmxaF*, MxaF knockout mutant. PCR confirmation of *mxaF* disruption using primers P1 and P2 **(B)**, P3 and P4 **(C)**. From genome sequence information for *M. trichosporium* OB3b, lengths of PCR amplicons using primers P1 and P2 from wild type and *ΔmxaF* mutant are expected to be 4.3 and 2.4 kbp, respectively **(B)**. PCR amplicons using primers P3 and P4 are expected to be 1.9 kbp from wild type only **(C)**. MxaF and XoxF immunodetection using specific antisera **(D)**.

We constructed a mutant of *ΔmxaF* in *M. trichosporium* OB3b using counterselection and double homologous recombination methods with *sacB* as a counterselectable marker (Figure 2A). We prepared the plasmids pJQ200SK_UpDw and pK18mobsacB_UpDw (Supplementary Figure S1). However, construction of the mutant succeeded only when pK18mobsacB_UpDw was used. Plasmid integration into the target site was confirmed by PCR with the primer sets A (mxaF_upst-F2 and mxaF_mid-R) and B (mxaF_dwst-R2 and mxaF_mid-F) on a single-crossover mutant grown on NMS with kanamycin (Supplementary Figure S2). The single-crossover mutant was plated on NMS containing sucrose and cerium for counterselection. We applied PCR to the surviving colonies and selected double-crossover mutants using primers P1 and P2. The PCR amplicons from the *ΔmxaF* mutants were shorter (2.4 kbp) than those from the wild type (4.3 kbp; Figure 2B). The PCR product was confirmed by sequencing. No PCR product from the *ΔmxaF* mutants was detected using primers P3 and P4 (Figure 2C). Hence, *mxaF* and the region derived from the integrated plasmid were successfully deleted. It was established that the mutant was resistant to sucrose but sensitive to kanamycin. Immunodetection with anti-MxaF and anti-XoxF antisera demonstrated the absence of MxaF expression and the presence of XoxF expression in the *ΔmxaF* mutant (Figure 2D). Thus, the targeted mutant without *mxaF* or the region from the integrated plasmid was successfully created.

### Cell Growth and Methanol Accumulation in the OB3b *ΔmxaF* Mutant

**Figure 3:**
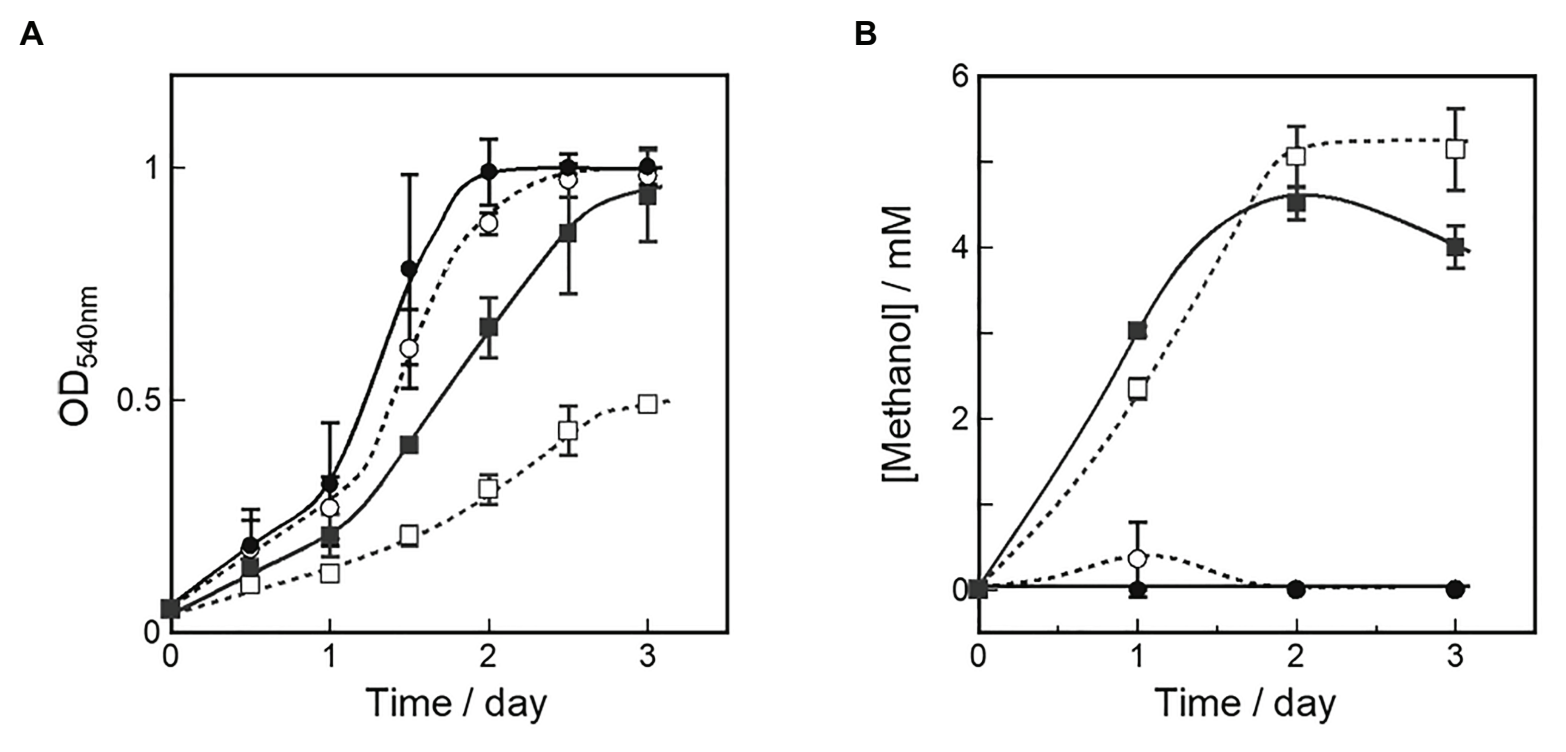
Cell growth **(A)** and methanol production **(B)** in OB3b *ΔmxaF* mutant in presence of various copper and cerium concentrations. A starter culture grown in NMS with 25 μM cerium ion was used as an inoculum to examine the growth and methanol production of the OB3b *ΔmxaF* mutant in the fresh medium of four different culture conditions. ○, 0 μM copper ion plus 0 μM cerium ion; ●, 0 μM copper ion plus 25 μM cerium ion; □, 10 μM copper ion plus 0 μM cerium ion; ■, 10 μM copper ion plus 25 μM cerium ion. Errors bars: duplicate sample ranges.

To evaluate our strategic design for methanol production and cell growth, we examined the OB3b *ΔmxaF* mutant construct in media with and without copper and cerium ions. We isolated the OB3b *ΔmxaF* mutant using selective growth media and incubated it in 20 ml NMS medium containing 25 μM cerium ion plus 0 μM copper ion. After OD_540_ = 0.8 was attained, the culture was washed twice with 8 ml fresh cerium‐ and copper-free NMS and transferred at a 1:9 ratio to NMS containing various copper and/or cerium ion concentrations. Figure 3A shows that the OB3b *ΔmxaF* mutant grew under all conditions but most slowly in the presence of 10 μM copper ion and the absence of cerium ion. According to the previous report, this growth of the OB3b *ΔmxaF* mutant can be caused by any remaining XoxF and cerium ion in the initial seed cells (Farhan Ul Haque et al., 2016). To evaluate whether the OB3b *ΔmxaF* mutants could be propagated, the cultures were inoculated into identical fresh media under the same conditions. When the mutants were transferred to identical fresh media at initial OD_540_ = 0.1 (transfer A), they grew in the presence of cerium ion (Supplementary Figure S3). However, the growth of the mutants was almost stopped in the absence of cerium ion. Therefore, this mutant required cerium ions to grow normally.

Methanol concentrations in the medium were measured simultaneously to evaluate methanol accumulation by the OB3b *ΔmxaF* mutant under each condition. Figure 3B shows that methanol accumulation was detected in all OB3b *ΔmxaF* mutants except those in the medium containing only 25 μM cerium ion plus 0 μM copper ion. Thus, methanol production with the OB3b *ΔmxaF* mutant can be realized by modulating the copper and cerium ion concentrations. Methanol production was also observed in the absence of an external electron donor. The methanol concentration was higher in the presence of 10 μM copper ion than it was in its absence. When the OB3b *ΔmxaF* mutant was cultured in medium containing 10 μM copper ion plus 0 μM cerium ion, the maximum methanol content was 2.6 μmol·mg^−1^ dry cell weight·h^−1^. One OD_540_ unit of *M. trichosporium* OB3b corresponds to 0.15 mg·ml^−1^ dry cell weight. Within 2 days, the methanol was saturated in the medium containing 10 μM copper ion plus 0 μM cerium ion. The conversion efficiency of the accumulated methanol to the total amount of methane added to the reaction system was ~0.3%. The methanol accumulation in the OB3b *ΔmxaF* mutant decreased with increasing incubation time except in the presence of 10 μM copper ion plus 0 μM cerium ion. The recovery of methanol accumulation was assessed in the OB3b *ΔmxaF* mutant *via* inoculation experiments under two conditions. After the cultures were transferred to identical fresh medium at the initial OD_540_ = 0.1 (transfer A), no methanol production was observed under any conditions (Supplementary Figure S3B). When the cultures were transferred to the same volume of identical fresh medium (transfer B; constant cell density), the methanol accumulation was maintained in the presence of 10 μM copper ion but stopped within 1 day after the medium was exchanged (Supplementary Figure S4). The methanol accumulation decreased on day 2 after the medium exchange in the presence of 10 μM copper ion plus 25 μM cerium ion. The cells were then replenished with identical fresh medium on day 5. Only slight methanol accumulation was detected in the medium containing 10 μM copper ion plus 0 μM cerium ion. Methanol accumulation was recovered by exchanging the medium but its activity nonetheless gradually declined thereafter. Though methanol accumulated in the presence of 25 μM cerium, there was an evident reduction in methanol concentration in the presence of 10 μM copper. Thus, XoxF consumed the methanol in the latter case.

**Figure 4:**
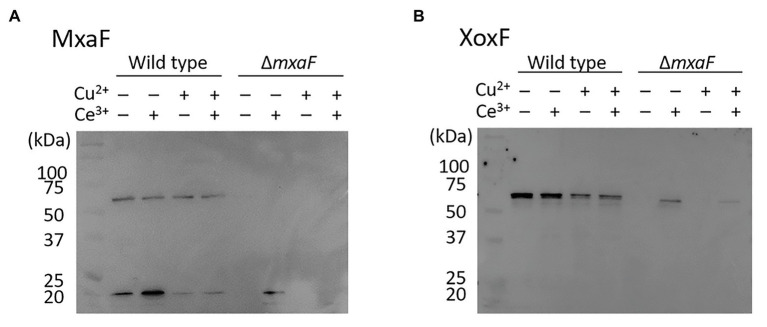
Western blot of wild type and *ΔmxaF* mutant of *M. trichosporium* OB3b. Immunodetection of MxaF **(A)** and XoxF **(B)** using specific antisera. Molecular weight marker (in kDa) of protein standards are shown on left.

We compared intracellular MDH expression by using *M. trichosporium* OB3b cultured in the medium under four different conditions. Western blotting of MDH showed that MxaF was absent in the OB3b *ΔmxaF* mutant under all conditions (Figure 4A). XoxF was expressed in the OB3b *ΔmxaF* mutant in the presence of cerium ion (Figure 4B). XoxF expression was higher in the absence than the presence of copper ion and the same held true for the OB3b wild type.

### Switching Between Cell Growth and Methanol Production in OB3b *ΔmxaF* Mutant

**Figure 5:**
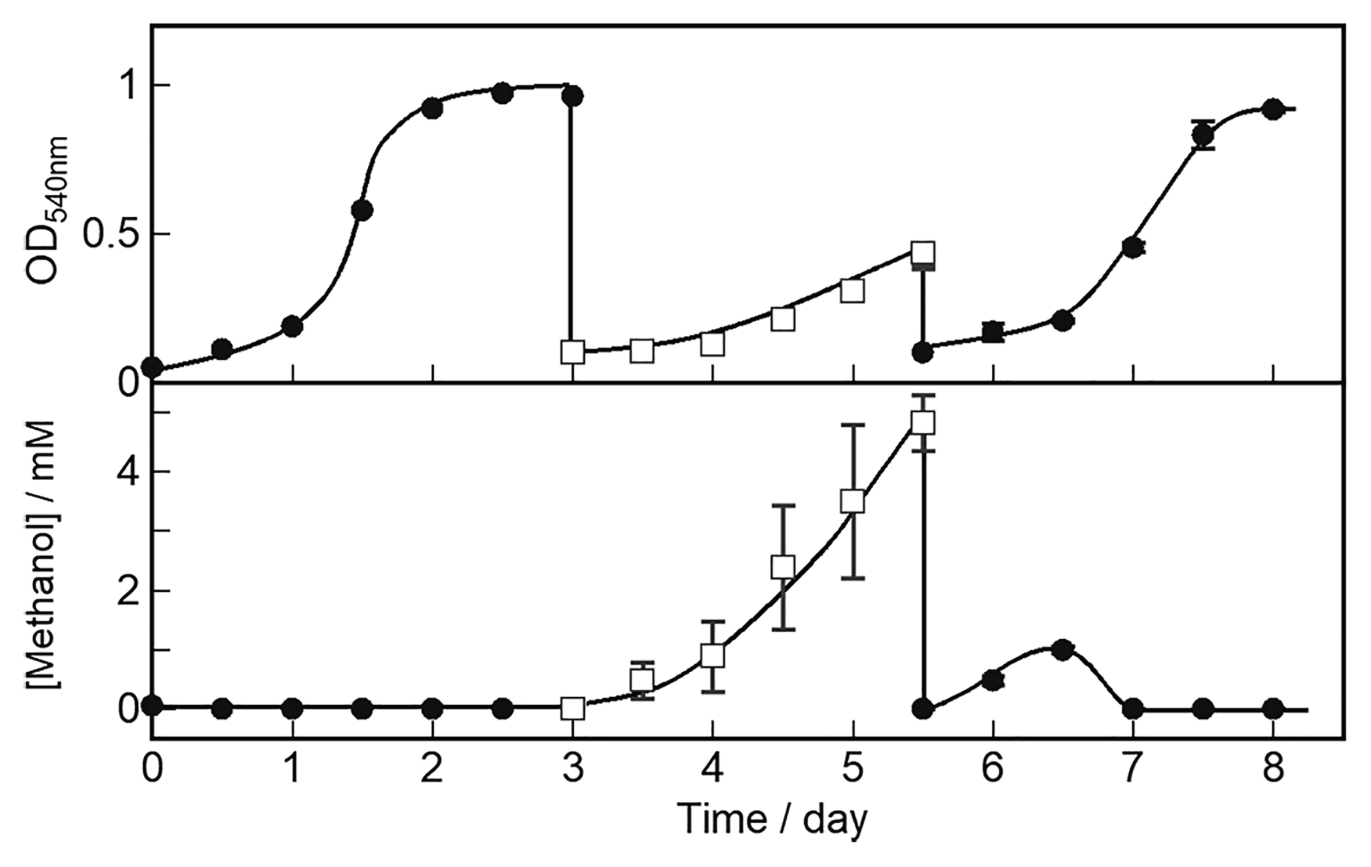
OB3b *ΔmxaF* mutant switching between cell growth and methanol production conditions by modulating copper and cerium concentrations. ●, cell growth condition (0 μM copper ion plus 25 μM cerium ion); □, methanol production condition (10 μM copper ion plus 0 μM cerium ion). Errors bars: duplicate sample ranges.

We characterized cell growth and methanol accumulation in the OB3b *ΔmxaF* mutant and demonstrated that its phenotype switched in response to changes in the metal ions in the medium. The cell growth curve and methanol concentration in the medium are shown in Figure 5. The OB3b *ΔmxaF* mutant was cultured with 0 μM copper ion plus 25 μM cerium ion. After 3 days, the cultures were collected, washed twice, and resuspended in NMS under conditions conducive to methanol production (10 μM copper ion plus 0 μM cerium ion) to obtain OD_540_ = 0.1 after inoculation (first switch). After 2.5 days, the cultures were collected, washed twice, and resuspended in NMS under cell growth conditions to obtain the initial OD_540_ = 0.1 (second switch). In the first switch, the growth rate of the OB3b *ΔmxaF* mutant decreased and methanol accumulation increased without the addition of any external electron source. The amount of methanol per unit cell (mg dry cell weight) increased until 1.5 days and then remained constant until 2.5 days (Supplementary Figure S5). In the second switch, the growth rate was restored to the same level as that of the first step. Methanol accumulation increased until day 1 but the methanol disappeared by day 1.5 when the cells entered the log phase. Therefore, cell growth was recovered because XoxF expression consumed methanol. The foregoing results disclosed that the OB3b *ΔmxaF* mutant alter its phenotype and XoxF MDH expression can be controlled by altering the medium such that it favors cell growth or methanol production.

## Discussion

The culture of whole-cell methanotrophs requires a strategic design to optimize both methanol production and cell growth. In the present study, we characterized the continuous cell growth and methanol production in the OB3b *ΔmxaF* mutant in nutrient media containing four different copper and cerium ion configurations (Table 3). We modulated the medium conditions to promote cell growth (0 μM copper ion plus 25 μM cerium ion) and methanol production (10 μM copper ion plus 0 μM cerium ion) and switch the OB3b *ΔmxaF* mutant phenotype. The mutant presented with nearly the same growth as the wild type under conditions conducive to cell growth and it expressed XoxF in the presence of cerium ion. Figure 3A shows that mutant grew under all conditions but had a longer lag phase in the presence than the absence of copper ion. Furthermore, its overall growth was slowest under conditions conducive to methanol production. For transfer A (Supplementary Figure S3), the OB3b *ΔmxaF* mutant grew best under cell growth conditions. Though it also grew in the presence of 10 μM copper and 25 μM cerium, its lag phase there was longer than it was under cell growth conditions. Cell growth was strongly inhibited when XoxF was not expressed and cerium ion was absent (Figure 4B). Mutant growth under each condition of the present study resembled that of another *M. trichosporium* OB3b *mxaF* knockout mutant reported by Muhammad Farhan Ul Haque et al. (2016). These authors also indicated a reduction in cell growth in the presence of copper ions and total cell growth arrest in the absence of cerium ions. Thus, the OB3b *ΔmxaF* mutant can grow using any remaining XoxF and cerium ion in the initial seed cells. It was shown that >98% of the added cerium is associated with microbial biomass (Farhan Ul Haque et al., 2015).

**Table 3:** Characterization of the continuous cell growth and methanol production in the OB3b *ΔmxaF* mutant under four conditions containing copper and cerium ions.

Metal ions in medium	MMO type	MDH type	Continuous cell growth	Methanol production
0 μM Cu^2+^ plus 0 μM Ce^3+^	sMMO	−	−	−
0 μM Cu^2+^ plus 25 μM Ce^3+^	sMMO	XoxF	++	−
10 μM Cu^2+^ plus 0 μM Ce^3+^	pMMO	−	−	++
10 μM Cu^2+^ plus 25 μM Ce^3+^	pMMO	XoxF	+	+

Methanol accumulation by the OB3b *ΔmxaF* mutant occurred in the medium containing copper ion. This condition is a requirement for pMMO expression (Figure 3B). Methane consumption was higher in whole-cell *M. trichosporium* OB3b expressing sMMO than it was in whole-cell *M. trichosporium* OB3b expressing pMMO (Sirajuddin and Rosenzweig, 2015). However, methanol did not accumulate in this mutant in the absence of copper and cerium ions which causes sMMO expression to predominate and represses XoxF (Figure 3B). Hence, methanol from sMMO was consumed more efficiently than methanol from pMMO by the remaining XoxF. The foregoing results and a previous study showed that pMMO forms a supercomplex with MxaF MDH (Culpepper and Rosenzweig, 2014). Coupling between pMMO and XoxF MDH is weaker than coupling between pMMO and MxaF MDH. Methanol may accumulate in cells expressing XoxF and pMMO because of weak coupling between XoxF and pMMO. XoxF does not oxidize methanol from pMMO as effectively as MxaF. This hypothesis explains the results shown in Figure 3B and Supplementary Figure S4. In the presence of 10 μM copper ion plus 25 μM cerium ion, the aforementioned mutant could accumulate methanol as it does under methanol production conditions. In the former scenario, however, it consumes any methanol generated thereafter. Despite pMMO and XoxF expression in the presence of 10 μM copper ion plus 25 μM cerium ion (Farhan Ul Haque et al., 2015, 2016), there was no methanol accumulation in the case of transfer A (Supplementary Figure S3). Methane oxidation by pMMO is evident in the presence of 10 μM copper ion plus 25 μM cerium ion. Hence, the mutant may regulate the balance between pMMO and XoxF and changes to the state appropriate for methane utilization. The methanol concentration reached saturation (~6 mM) in the OB3b *ΔmxaF* mutant grown in medium containing 10 μM copper ion plus 0 μM cerium ion (Figure 3B). Methanol accumulation was arrested as methanol inhibited pMMO activity reversibly (Furuto et al., 1999). The observed recovery of methanol accumulation in the OB3b *ΔmxaF* mutant after the fresh medium was exchanged corroborated this hypothesis (Supplementary Figure S4). One way to overcome this limitation is to use a continuous culture system wherein the medium is exchanged constantly and the methanol is removed.

To the best of our knowledge, this study is the first to report methanol production using a genetically modified methanotroph lacking MDH activity. Changing the phenotype of this mutant by altering the metal ion concentrations in its growth medium facilitates the recovery of bacterial enzymes, reducing equivalents, and methanol production. Therefore, this mutant can function as a methanol production biocatalyst for longer periods of time than conventional methods. A previous study on methanol production by methanotrophs revealed that several MDH inhibitors have been implemented to stop methanol metabolism. Furuto et al. (1999) reported semicontinuous methanol synthesis with *M. trichosporium* OB3b in the presence of the MDH inhibitor cyclopropanol. The methanol production rate was 3.2 μmol·mg^−1^ cell·h^−1^ after the addition of 14.3 mM sodium formate. Methanol production rates of 6.0 μmol·mg^−1^ cell·h^−1^ (Mehta et al., 1987) and 2.6 μmol·mg^−1^ cell·h^−1^ (In Yeub et al., 2015) were reported for *M. trichosporium* OB3b. The methanol production rate for *M. trichosporium* OB3b mutant in this study (2.6 μmol·mg^−1^ cell·h^−1^) was similar to those of previous reports. The mutant had the same methanol production capacity as the wild type subjected to MDH inhibitors. However, the OB3b *ΔmxaF* mutant requires time to switch between cell growth and methanol production. After its phenotype was switched by changing the growth medium, the former characteristics of the mutant were restored for ~1 day before the phenotype transitions occurred (Figure 5). This lag time may be explained by the persistence of XoxF MDH and/or pMMO from the previous state in the medium. One day after modulating the metal ion composition of the medium, the characteristics of the alternate phenotype may be observed in most cells under the new condition. Therefore, strategies are required to improve the efficacy of the switching system to reduce the lag time in the phenotypic change. In addition, the low methanol conversion efficiency for *M. trichosporium* OB3b mutant in this study (~0.3% of the total amount of methane added to the reaction system) is an issue that needs to be improved. In recent years, new bioreactors that are effective for methanotrophs have been reported (Chen et al., 2020; Valverde-Pérez et al., 2020). It is expected that more efficient carbon conversion can be achieved by using these bioreactors.

Here, the *M. trichosporium* OB3b mutant of mxaF knockout (OB3b *ΔmxaF* mutant) was constructed by a double homologous recombination method and *sacB* was used as a counterselectable marker. The OB3b *ΔmxaF* mutant normally grows by expressing XoxF MDH in media containing cerium ion. It accumulates methanol by expressing pMMO in media containing copper ion and no external metabolic electron donors. The phenotype of the OB3b *ΔmxaF* mutant can be altered between that which is found under cell growth conditions and that which occurs under methanol production conditions by changing the cerium and copper ion content in the growth media. Phenotype switching in the OB3b *ΔmxaF* mutant achieves continuous methanol production by restoring reducing power. The present study proposes an innovative strategy for methanol production using genetically engineered, metabolically modified methanotrophs. Although it is not yet cost-effective for industrial methanol production, this study shows a new possibility for methanol production using methanotrophs. Subsequent research should focus on optimizing the medium conditions and culture system under which the mutant could serve as an industrial biocatalyst converting methane to methanol.

## Data Availability Statement

The raw data supporting the conclusions of this article will be made available by the authors, without undue reservation.

## Author Contributions

HI and KY conducted the experiments. MI and KH assisted in the experiment design and gave a hand in performing the experiments. HI and TK analyzed the data and wrote the manuscript. All authors contributed to the article and approved the submitted version.

### Conflict of Interest

The authors declare that the research was conducted in the absence of any commercial or financial relationships that could be construed as a potential conflict of interest.

## Supplementary Material

Supplementary Figure S1 Plasmid construction protocol. **(A)** pJQ200SK_UpDw, **(B)** pK18mobsacB_UpDw. Suicide plasmids used *Gm^r^* (gentamicin resistance gene), *Km^r^* (kanamycin resistance gene), and *sacB* as counterselectable markers.

Supplementary Figure S2 PCR analysis of single crossover strains in genomic DNA of *M. trichosporium* OB3b::pK18mobsacB_UpDw using primer sets A (mxaF_upst-F2 and mxaF_mid-R) and B (mxaF_dwst-R2 and mxaF_mid-F). **(A)** Outline of experiment. **(B)** Electrophoresis of colony PCR amplicon using primer sets A and B. M, marker; WT, wild type. Red square: PCR product of recombination between downstream regions. Blue square: PCR product of recombination between upstream regions.

Supplementary Figure S3 Cell growth and methanol accumulation in OB3b Δ*mxaF* mutant after subculture with identical media at initial OD_540_ = 0.1. ○, 0 μM copper ion plus 0 μM cerium ion; ●, 0 μM copper ion plus 25 μM cerium ion; □, 10 μM copper ion plus 0 μM cerium ion; ■, 10 μM copper ion plus 25 μM cerium ion.

Supplementary Figure S4 Continuous methanol production using OB3b Δ*mxaF* mutant in presence of various copper and cerium concentrations. ○, 0 μM copper ion plus 0 μM cerium ion; ●, 0 μM copper ion plus 25 μM cerium ion; □, 10 μM copper ion plus 0 μM cerium ion; ■, 10 μM copper ion plus 25 μM cerium ion. Exchange of identical fresh media containing all cultures on days 3 and 5.

Supplementary Figure S5 OB3b *ΔmxaF* mutant switching between cell growth and methanol production conditions by modulating copper and cerium concentrations. One OD_540_ unit of *M. trichosporium* OB3b corresponds to 0.15 mg·ml^-1^ dry cell weight. ●, cell growth condition (0 μM copper ion plus 25 μM cerium ion); □, methanol production condition (10 μM copper ion plus 0 μM cerium ion). Errors bars: duplicate sample ranges.
