# Supplementary figures and images for: Switching Between Methanol Accumulation and Cell Growth by Expression Control of Methanol Dehydrogenase in Methylosinus trichosporium OB3b Mutant
Source: Front Microbiol. 2021 Mar 22;12:639266. doi: 10.3389/fmicb.2021.639266 (PMC8019695; doi:10.3389/fmicb.2021.639266)

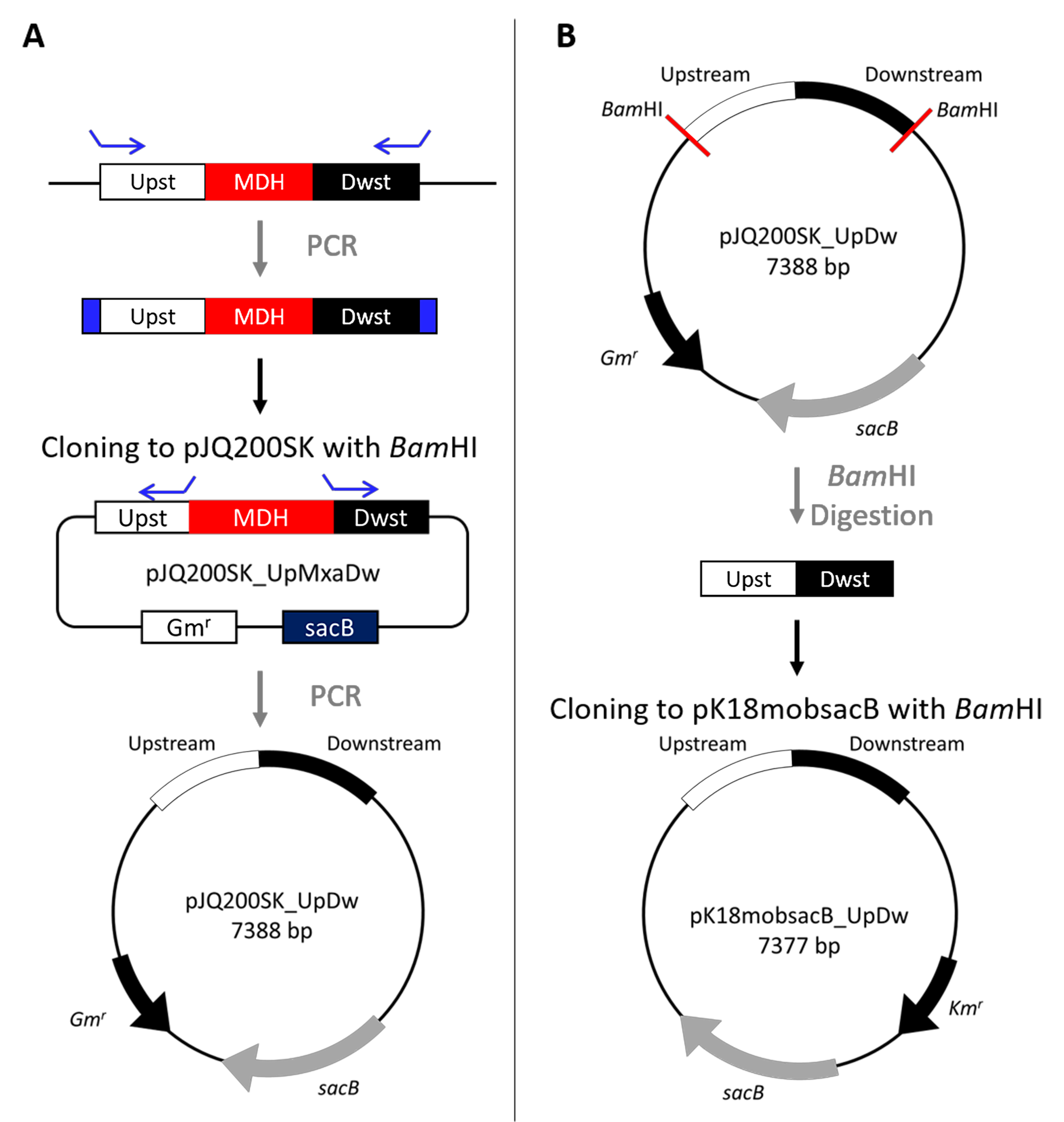

Supplement: Supplementary Figure S1 — Plasmid construction protocol. (A) pJQ200SK_UpDw, (B) pK18mobsacB_UpDw. Suicide plasmids used Gmr (gentamicin resistance gene), Kmr (kanamycin resistance gene), and sacB as counterselectable markers. [file Image_1.tif]

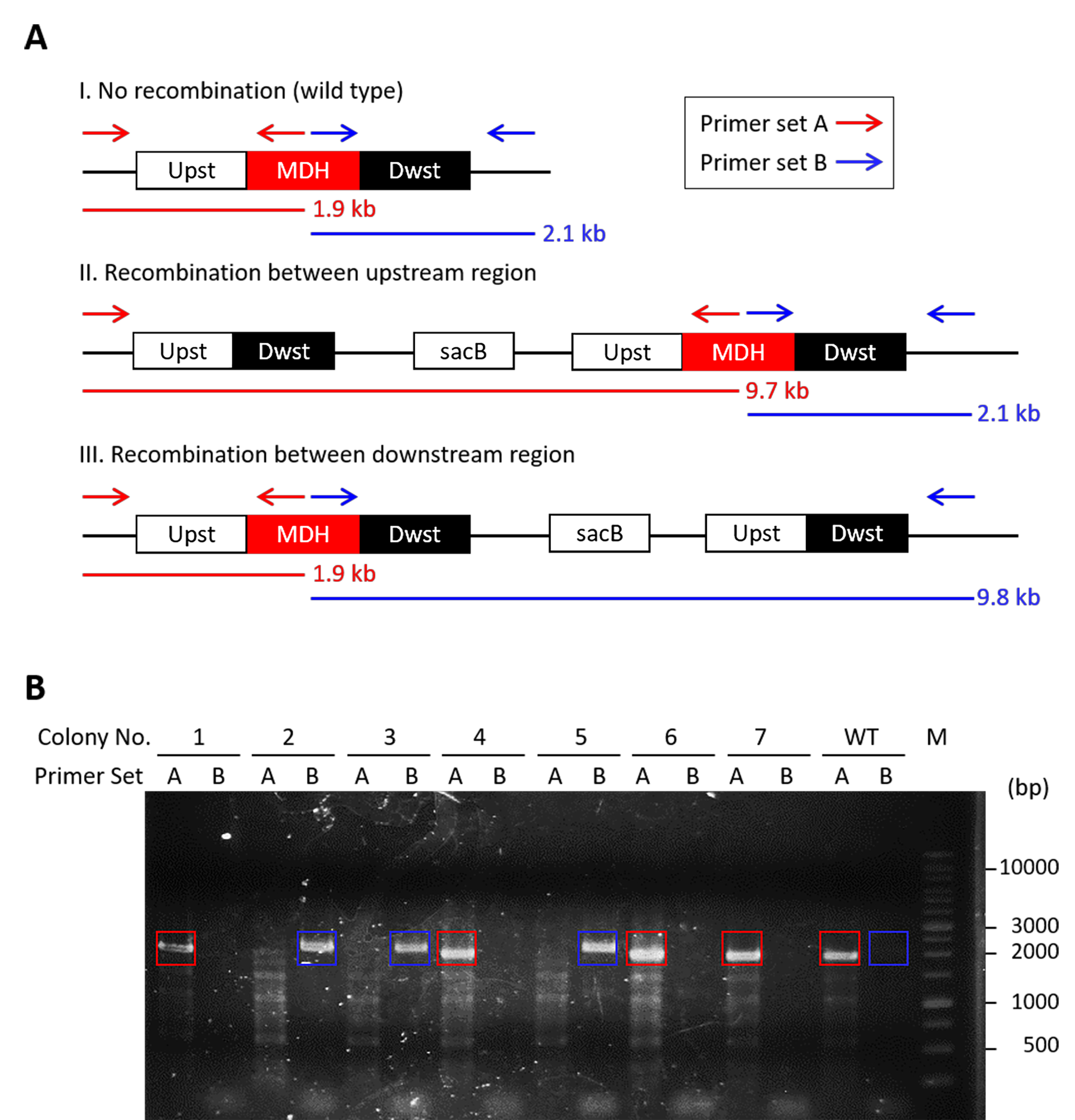

Supplement: Supplementary Figure S2 — PCR analysis of single crossover strains in genomic DNA of M. trichosporium OB3b::pK18mobsacB_UpDw using primer sets A (mxaF_upst-F2 and mxaF_mid-R) and B (mxaF_dwst-R2 and mxaF_mid-F). (A) Outline of experiment. (B) Electrophoresis of colony PCR amplicon using primer sets A and B. M, marker; WT, wild type. Red square: PCR product of recombination between downstream regions. Blue square: PCR product of recombination between upstream regions. [file Image_2.tif]

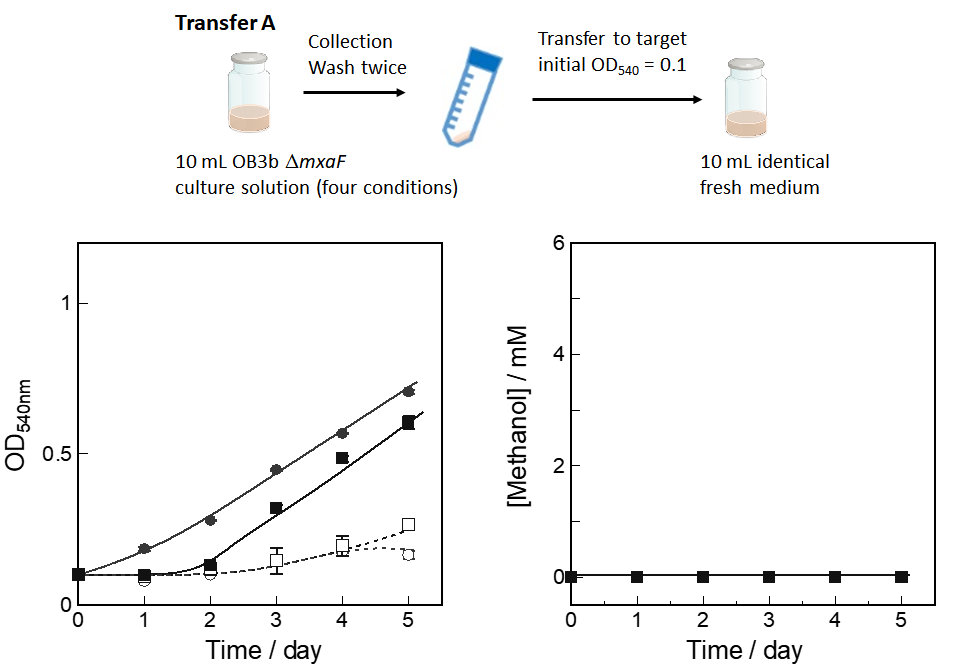

Supplement: Supplementary Figure S3 — Cell growth and methanol accumulation in OB3b ΔmxaF mutant after subculture with identical media at initial OD540 = 0.1. ○, 0 μM copper ion plus 0 μM cerium ion; ●, 0 μM copper ion plus 25 μM cerium ion; □, 10 μM copper ion plus 0 μM cerium ion; ■, 10 μM copper ion plus 25 μM cerium ion. [file Image_3.tif]

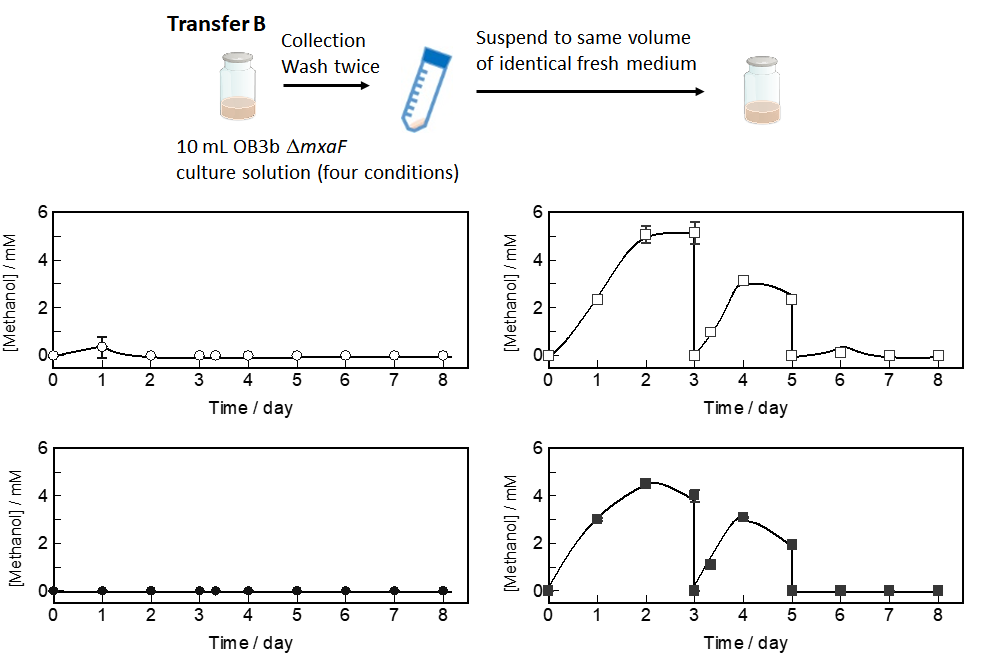

Supplement: Supplementary Figure S4 — Continuous methanol production using OB3b ΔmxaF mutant in presence of various copper and cerium concentrations. ○, 0 μM copper ion plus 0 μM cerium ion; ●, 0 μM copper ion plus 25 μM cerium ion; □, 10 μM copper ion plus 0 μM cerium ion; ■, 10 μM copper ion plus 25 μM cerium ion. Exchange of identical fresh media containing all cultures on days 3 and 5. [file Image_4.tif]

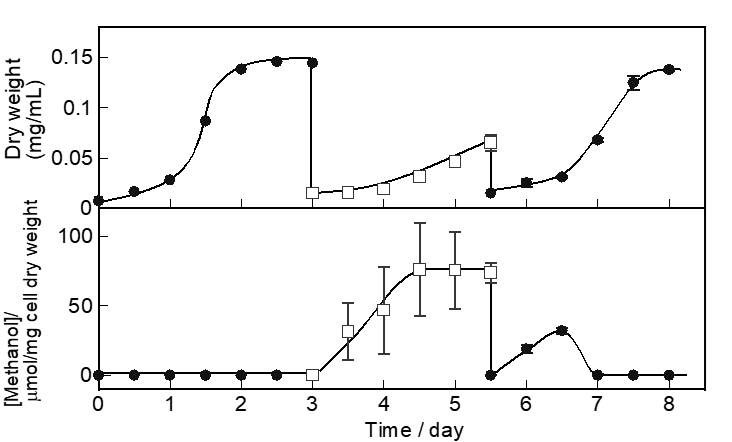

Supplement: Supplementary Figure S5 — OB3b ΔmxaF mutant switching between cell growth and methanol production conditions by modulating copper and cerium concentrations. One OD540 unit of M. trichosporium OB3b corresponds to 0.15 mg·ml-1 dry cell weight. ●, cell growth condition (0 μM copper ion plus 25 μM cerium ion); □, methanol production condition (10 μM copper ion plus 0 μM cerium ion). Errors bars: duplicate sample ranges. [file Image_5.tif]
